# Endovascular management of a fractured dialysis catheter: a case report and review of retrieval techniques

**DOI:** 10.3389/fsurg.2025.1700677

**Published:** 2025-11-12

**Authors:** Tianjun Lin, Qiqi Wang, Wei Huang, Yang Liu, Chunshui He

**Affiliations:** 1 Hospital of Chengdu University of Traditional Chinese Medicine, Chengdu, China; 2 Department of Vascular Surgery, Hospital of Chengdu University of Traditional Chinese Medicine, Chengdu, China

**Keywords:** hemodialysis catheter, intravascular foreign body, endovascular retrieval, interventional nephrology, case report

## Abstract

**Background:**

With the advancements in endovascular techniques, reports of rare vascular complications have increased. This case describes the accidental fracture of a non-cuffed hemodialysis catheter and its subsequent endovascular management, offering valuable insights for the treatment of similar intravascular foreign bodies (IFBs).

**Case report:**

An 83-year-old male with a non-cuffed catheter presented after a catheter fracture. Radiographic imaging confirmed migration of the fragment to the right atrium. After a multispecialistic collaboration assessment, endovascular retrieval was performed via right femoral access using a filter retrieval device under fluoroscopic guidance. The catheter fragment was successfully captured and removed without procedural complications. The patient recovered uneventfully and remained well at follow-up evaluations.

**Conclusion:**

This case highlights the efficacy and safety of percutaneous endovascular retrieval for managing IFBs, providing a minimally invasive alternative to surgery with high success rates. Meticulous catheter handling and regular integrity assessments are critical to prevent and promptly manage such events.

## Introduction

The growing dependence on non-cuffed catheters for renal replacement therapy has been accompanied by an increase in associated mechanical complications. Catheter fracture, which may result from excessive traction, accidental damage from sharp objects (1), or material fatigue and degradation (2), constitutes a rare yet potentially life-threatening event that requires urgent diagnosis and intervention. Determining the optimal management strategy for such complications remains a critical clinical challenge, necessitating a careful balance between the risks of interventional procedures and the hazards posed by a retained intravascular foreign body. This report describes a rare case of a fractured non-cuffed catheter that migrated to the right atrium and was successfully retrieved using a minimally invasive endovascular technique. We seek to share pertinent insights and clinical experiences for clinicians encountering similar cases.

## Case presentation

An 83-year-old male with chronic renal failure presented to the emergency department. Three weeks ago, the patient had undergone creation of a right forearm arteriovenous graft (AVG) and placement of a 12 Fr-16 cm non-cuffed catheter (DIALL Medical Technology Co., Ltd., Zhengzhou, China) in the right internal jugular vein for continuous renal replacement therapy (CRRT), following thrombosis of his autogenous arteriovenous fistula. After discharge, he had been receiving regular dialysis sessions as scheduled.

**Figure 1:**
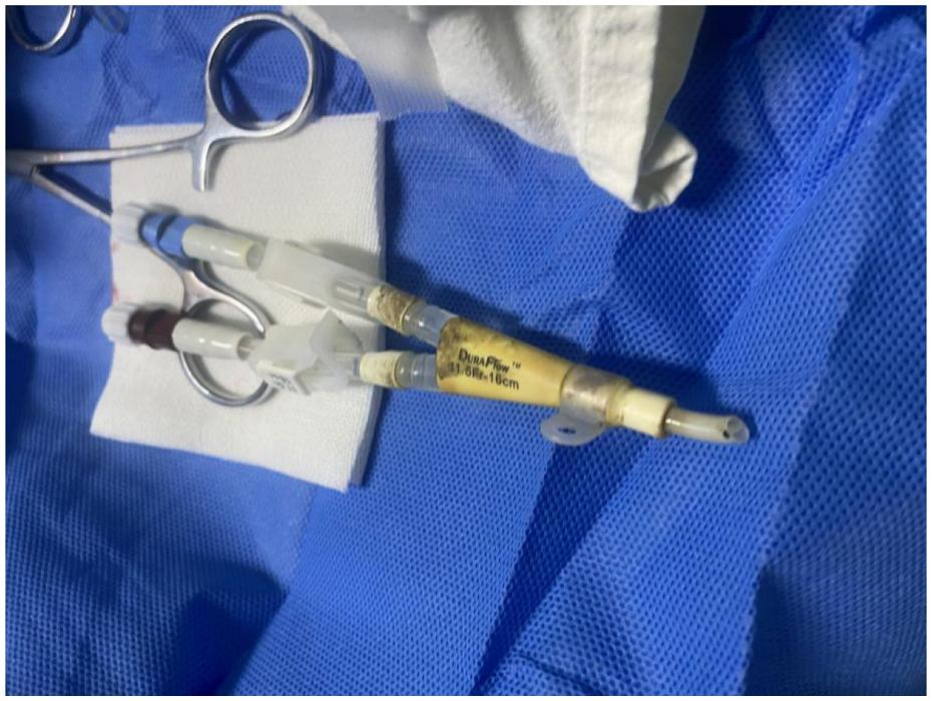
Fractured catheter fragment retained externally.

**Figure 2:**
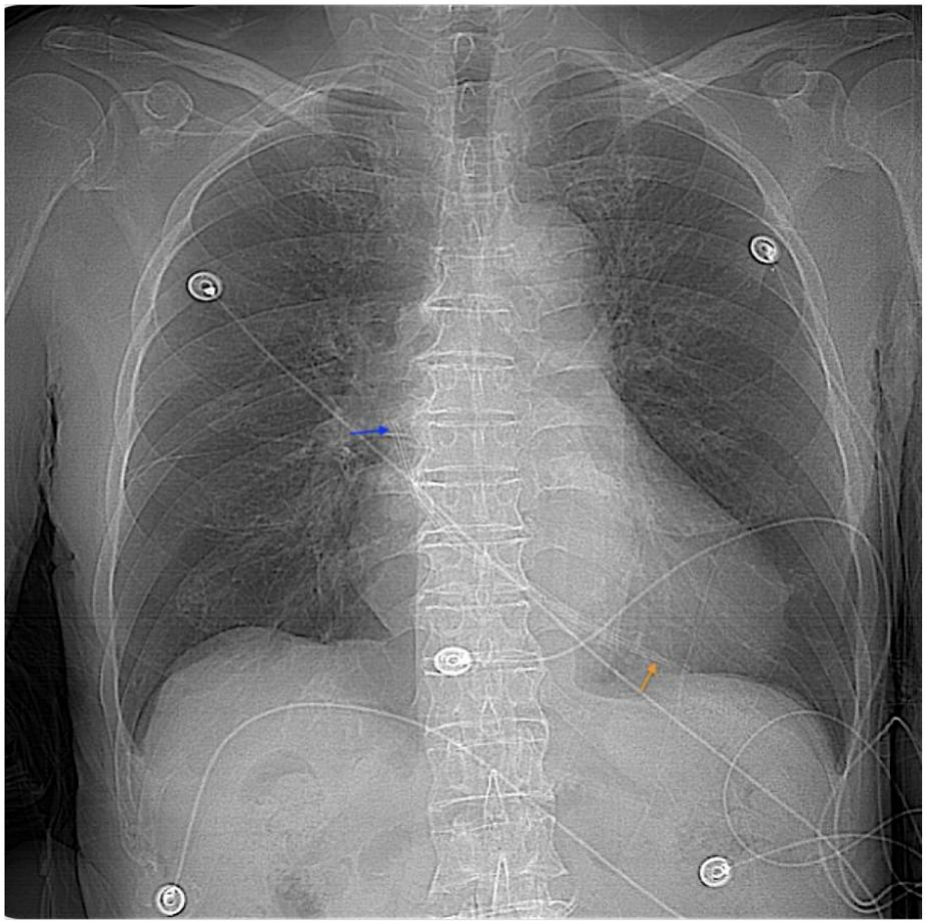
CT fluoroscopy showing the temporary dialysis catheter located in the atrium. Orange arrow point to the catheter tip, and the blue arrow point to the tail.

At 5:00 AM on the day of admission, the dialysis catheter fractured. The patient, along with family members who brought the fractured catheter segment, presented to the emergency room. The patient was applying continuous manual compression to the right neck. Physical examination by the emergency physician revealed no visible remnant of the catheter at the insertion site; the whereabouts of the remaining fractured segment was unknown (Figure 1), and there was no active bleeding from the wound. An emergent chest CT scan revealed the fractured fragment to be entirely intravascular (Figure 2), with its tip located in the atrium and no evidence of a subcutaneous component.

Following a multidisciplinary consultation involving nephrology, anesthesiology, and cardiothoracic surgery, and after a comprehensive assessment of the patient's overall condition—including cardiac function and coagulation status—to minimize procedural risks, both open surgical intervention and conservative management were ruled out. After obtaining informed consent from the patient and his family, we decided to proceed with percutaneous catheter retrieval as the treatment of choice.

**Figure 3:**
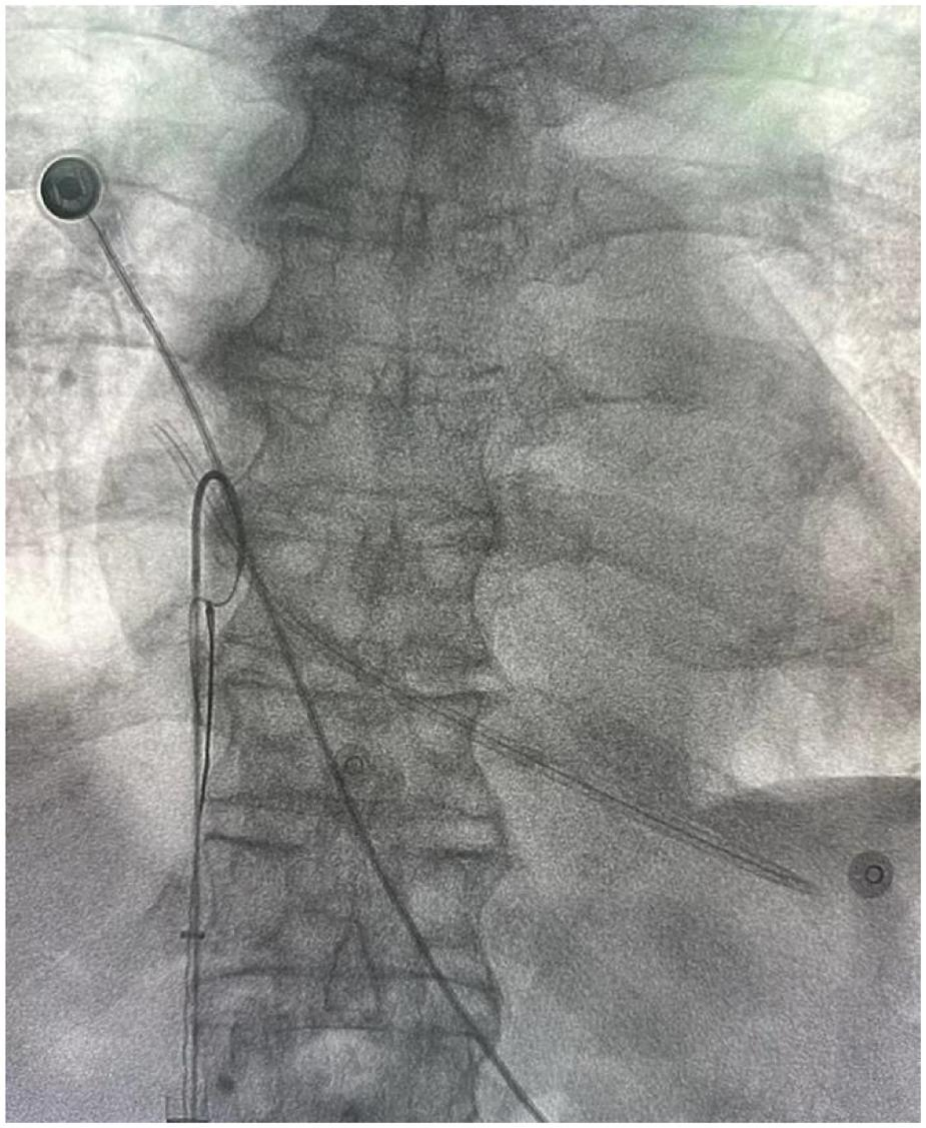
Filter retrieval device combined with a pigtail catheter to attempt capture of the fractured dialysis catheter.

We urgently transferred the patient to the interventional suite for the procedure. We initiated intraprocedural anticoagulation prophylaxis with low-molecular-weight heparin (LMWH) at a dose of 50 IU/kg. After administering local anesthesia to the groin region, we punctured the right common femoral vein under ultrasound guidance using the Seldinger technique and inserted a 6Fr vascular sheath (Cordis, Miami Lakes, FL, USA). We then advanced a 0.035-inch hydrophilic guidewire (Terumo, Somerset, NJ, USA) and a pigtail catheter (Cordis) into the superior vena cava near the cardiac region. Portable C-arm fluoroscopy confirmed that the fractured catheter segment traversed the superior vena cava and extended into the cardiac chamber (Figure 3).

**Figure 4:**
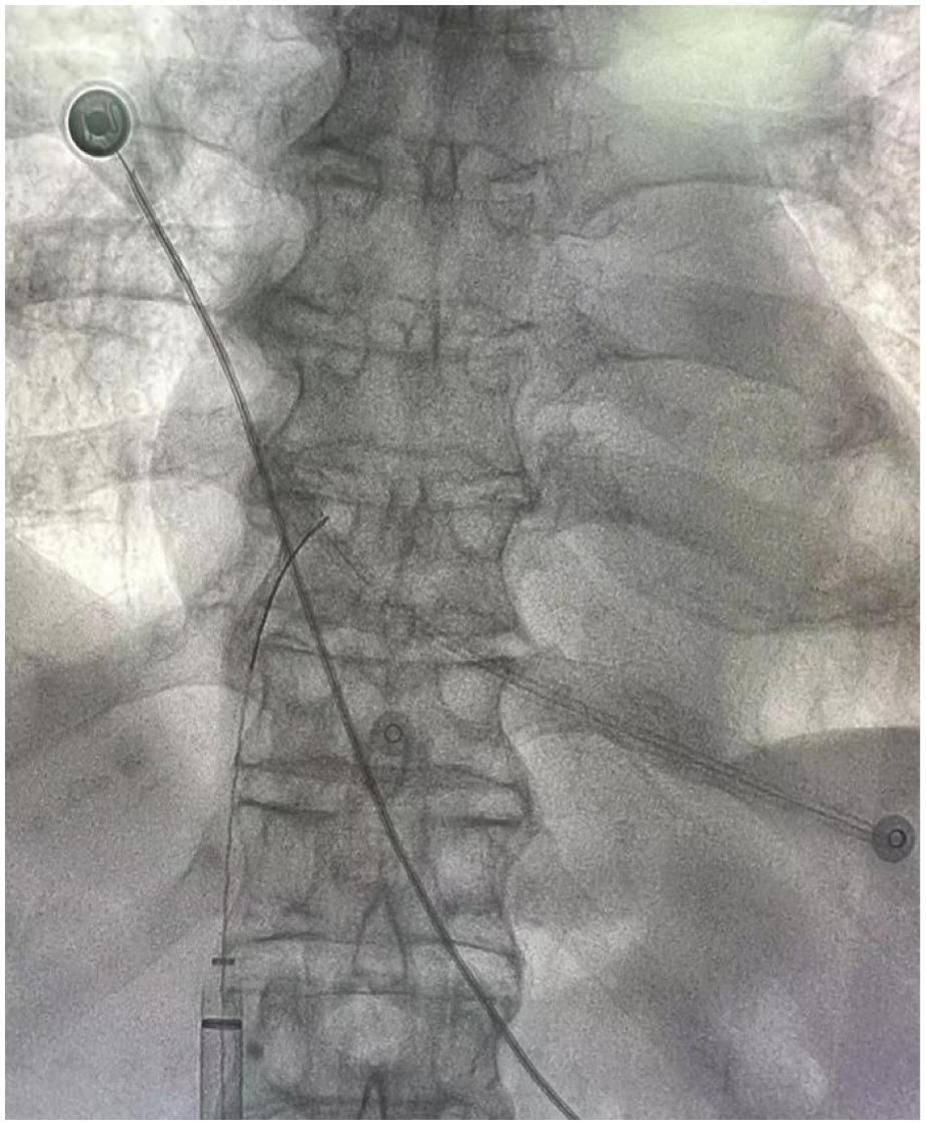
Filter retrieval device successfully snares the distal of the catheter.

**Figure 5:**
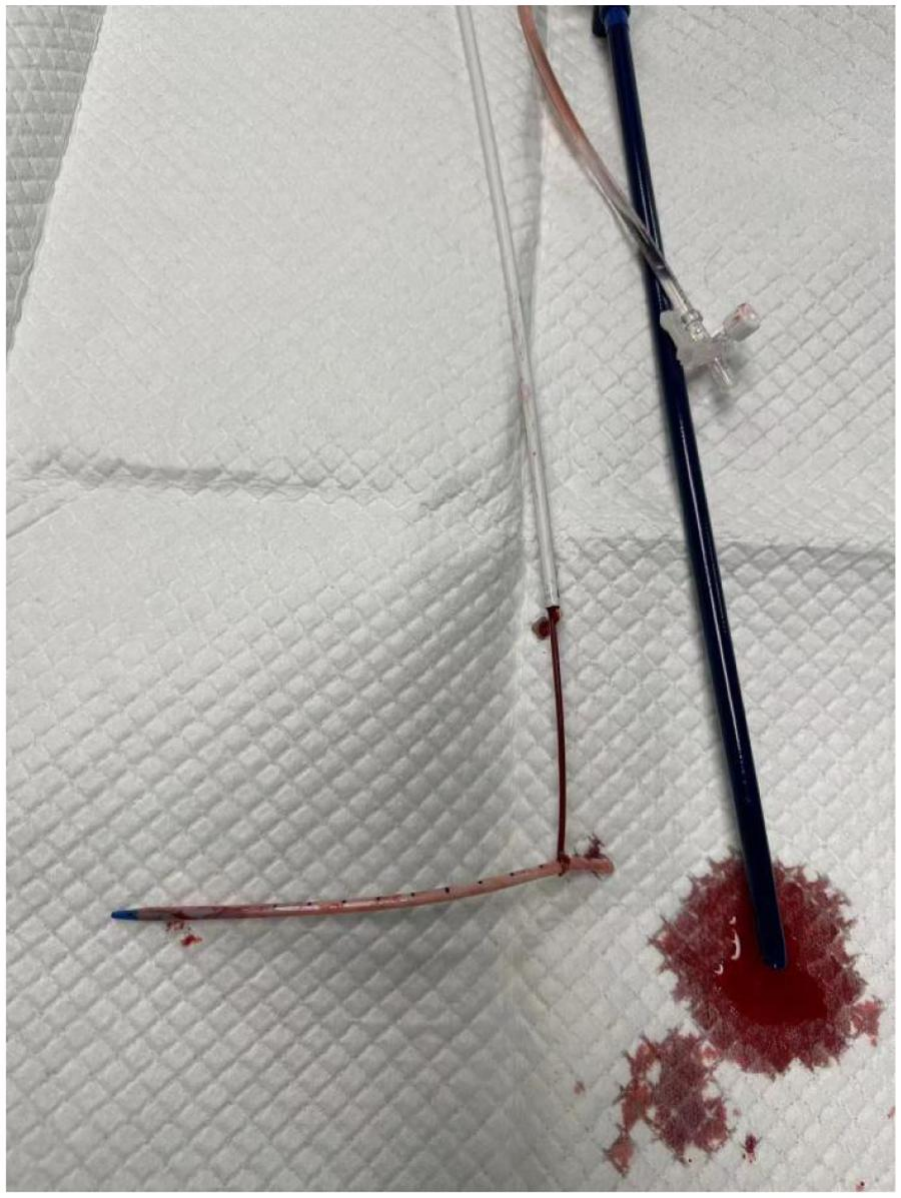
Successful retrieval of the retained catheter segment.

Under continuous fluoroscopic guidance, we deployed a filter retrieval device (Bard Peripheral Vascular Inc., Minnesota, USA) and accurately captured the proximal end (the most accessible segment) of the fractured catheter. After confirming secure engagement of the foreign body (Figure 4), we gradually and carefully retracted the captured catheter into the introducer sheath to avoid vascular wall injury or further migration of the fragment. We then successfully withdrew the catheter into the retrieval device and removed it entirely from the body (Figure 5).

The retrieved catheter fragment measured approximately 13 cm in length. We immediately performed post-procedural fluoroscopy to confirm the complete removal of all fragments and documented the length of the retrieved foreign body. We compared this measurement with the preoperative CT assessment to ensure no residual material remained. The entire procedure was uneventful. Post-operatively, the patient was closely monitored and exhibited no complications such as arrhythmia, thrombosis, or puncture site hemorrhage. During the 3-day hospitalization, the patient received continued anticoagulation with LMWH (14,000 IU daily; SANOFI WINTHROP INDUSTRIE). A new non-cuffed catheter was inserted into the left internal jugular vein, followed by one session of hemodialysis. The patient and his family also received one session of psychological counseling to alleviate postoperative anxiety. Follow-up duplex ultrasonography at one and three months postprocedure revealed no thrombosis in the right internal jugular vein, and the patient recovered satisfactorily.

## Discussion

With the rising incidence of chronic kidney disease (CKD), the number of patients receiving continuous renal replacement therapy (CRRT) using temporary dialysis catheters has also been increasing. Although catheter fracture remains a rare complication, its occurrence can lead to severe clinical consequences and requires prompt recognition and management. This study reports a rare and severe complication in which a fractured non-cuffed dialysis catheter migrated into the right atrium and was successfully retrieved using a percutaneous endovascular approach. This case demonstrates the critical role of multispecialistic collaboration and endovascular techniques of IFBs. Furthermore, it offers practical insights that may assist clinicians in the management of comparable high-risk scenarios.

Common causes of catheter fracture include material fatigue from repeated compression or twisting, traumatic traction, accidental damage by sharp objects, and degradation of the catheter material over time. In this case, the fracture may have resulted from repeated patient manipulation or accidental trauma with a sharp object—potentially associated with underlying psychological distress—leading to catheter fragmentation. This highlights the importance of enhancing psychological assessment and support for patients undergoing long-term dialysis. Studies have indicated that adequate social support can significantly improve treatment adherence and quality of life, while alleviating anxiety and depressive symptoms (3, 4).

In recent years, with advances in interventional therapy techniques, there has been a corresponding increase in reports of fractured guidewires (5), particularly among hemodialysis and oncology patients (6–8). Retained guidewires are reportedly associated with a mortality rate as high as 20% (9). The risk of complications associated with retained intravascular foreign bodies (RIFBs) rises with prolonged dwell time, as does the technical challenge of retrieval (10). Therefore, early diagnosis and prompt removal of RIFBs are crucial.

Preoperative multidisciplinary consultation is imperative in cases where the extent of IFBs remains unclear. Such consultation aids in formulating treatment strategies, anticipating potential complications, selecting the most appropriate surgical approach, and planning postoperative care—highlighting the critical role of multispecialistic collaboration. Currently, x-ray serves as the primary modality for detecting retained or fractured guidewires or catheter fragments. However, it is limited to projective localization and cannot provide precise anatomical spatial relationships (11). In contrast, CT/CTA offers more accurate spatial localization of IFBs compared to x-ray, particularly in visualizing small fragments such as fractured guidewires (12, 13). Nevertheless, CTA may exhibit lower sensitivity than DSA for detecting non-metallic IFBs, such as plastic catheter fragments (14). Therefore, high-resolution DSA is recommended for its superior real-time imaging capabilities during endovascular procedures, which significantly enhance precision in retrieval operations (15). The availability of an experienced vascular surgery team and a well-equipped facility is also crucial for optimizing success rates in the management of these complex cases.

Endovascular intervention has now emerged as the preferred strategy for the management of intravascular foreign bodies (IFBs), owing to its minimally invasive nature, high procedural success, expedited recovery, and the preservation of future treatment options (16, 17). In the retrieval of IFBs, gooseneck loop snares are the most commonly used devices for percutaneous removal (18). In this case, via a femoral venous approach and under DSA guidance, the proximal end of the catheter was successfully captured using a gooseneck snare and completely removed without vascular injury or other complications. This procedure fully demonstrates the efficacy and safety of interventional techniques in managing foreign bodies in the central vascular region. Nonetheless, open surgical intervention remains necessary in approximately 6% of cases, particularly when IFBs are embedded, entangled, or adjacent to critical anatomical structures (19). Therefore, treatment selection should be individualized, taking into account the characteristics of the foreign body, its location, and the overall clinical condition of the patient.

For example, Marco Coli et al. (20) reported a Swan-Ganz catheter was inadvertently placed into the left carotid artery and formed a knot at its tip. After an unsuccessful endovascular retrieval attempt, the catheter was eventually removed through a left carotid artery cutdown. Similarly, Sharma (21) described a fractured dialysis catheter that had been inserted via the femoral vein and became lodged near the junction of the right external and internal iliac veins; the fragment was subsequently extracted via open surgical venotomy. Ribeiro (22) reported five cases of IFBs removal, two of which involved the retrieval of fractured dialysis catheters using a combined approach of femoral venotomy and endovascular technique. All these reported cases ultimately required surgical intervention, which carries risks such as significant trauma, wound infection, and extended operative time. In contrast, the catheter in this case was situated in the cardiac region and exhibited a relaxed, non-entangled configuration, rendering it amenable to complete endovascular retrieval without surgical intervention.

As shown in Table 1, some patients received endovascular retrieval of the fractured catheter, while others required surgical. The selection of the appropriate technique—endovascular, surgical, or hybrid—is influenced by multiple factors. These include the location and course of the fractured catheter, the presence of kinking or entanglement, and the patient's vascular anatomy. Although percutaneous endovascular retrieval is a minimally invasive strategy and offers distinct advantages over surgical, specific scenarios may necessitate surgical intervention to ensure complete removal and minimize the risk of complications. In this case, the fractured catheter segment is in the superior vena cava and cardiac chamber, with no evidence of kinking or complex entanglement. Given the favorable anatomical conditions and the substantial expertise of our vascular surgery team, we selected endovascular retrieval as the primary approach. We performed the procedure successfully, and the patient recovered well, with no complications observed at one- and three-month follow-up examinations.

**Table 1:** Characteristics of cases of dialysis catheter rupture reported in the literature.

Author(s)	Age/sex	Foreign Body	Implantation site	Location Site	Treatment modality	Device	Outcome
Felipe Soares Ribeiro et al.	57/Female	Hemodialysis catheter	Right jugular vein	SVC/RA	Inguinotomy + GSV puncture	Pigtail	Retrieval
	55/Male	Hemodialysis catheter	Right jugular vein	SVC/RA	Inguinotomy + GSV puncture	Pigtail	Retrieval
Su Nam Lee et al.	44/Male	Hemodialysis catheter	Rright subclavian vein	SVC	Inguinotomy + RFV puncture	balloon	Retrieval
Pei-Jun Li et al.	61/Female	Hemodialysis catheter	Right jugular vein	SVC/RA	RFV puncture	Gooseneck loop snares	Retrieval
Tomoko Sasaki et al.	75/Female	The tunneled cuffed hemodialysis catheter	Right jugular vein	RA	RFV puncture	triple-loop snare	Retrieval
Ugo Vertolli et al.	70/Female	Hemodialysis catheter	Rright subclavian vein	RV	No treatment	None	no clinical issues in RV
Aditya Sharma et al.	58/Male	non-tunnelled temporary hemodialysis catheter	Right femoral vein	EIV/IIV	Inguinotomy	None	Retrieval
Huizhen Wu et al.	58/Female	temporary hemodialysis catheter	Right jugular vein	IJV	phlebotomy	None	Retrieval
Anand Reddy et al.	35/Female	CVC	Right jugular vein	RA	RFV puncture	percutaneous snare	Retrieval

GSV, great saphenous vein; RA, right atrium; RV, right ventricle; RFV, right femoral vein; SVC, superior vena cava; EIV, external iliac vein; IIV, internal iliac vein; IJV, internal jugular vein; CVC, central venous catheter.

## Conclusion

This case report highlights the clinical significance of non-cuffed catheter fracture as a rare but serious complication and emphasizes the pivotal role of percutaneous endovascular techniques in the retrieval of intravascular foreign bodies (IFBs). In comparison to open surgical, endovascular intervention offers minimal invasiveness, high technical success, and rapid recovery, establishing it as the first-line treatment when anatomically feasible.

To mitigate such complications, dialysis catheters should be meticulously secured and removed exclusively under the supervision of trained and certified clinicians. Furthermore, careful inspection of catheter integrity—particularly verification of its complete length upon removal—is imperative. Any discrepancy in catheter length should be promptly communicated to both interventional radiology and vascular surgery teams to enable immediate diagnostic assessment and intervention.

## Data Availability

The original contributions presented in the study are included in the article/Supplementary Material, further inquiries can be directed to the corresponding author.
